# Supplementary material for: Structural Mechanism and Cellular Restriction of Tau Seeding from Endolysosomes
Source: bioRxiv. 2026 Jun 10:2026.06.08.731026. Preprint. [Version 1] doi: 10.64898/2026.06.08.731026 (PMC13277937; doi:10.64898/2026.06.08.731026)
Supplement: Supplementary file 1 [file NIHPP2026.06.08.731026v1-supplement-2.pdf]

bioRxiv preprint doi: https://doi.org/10.64898/2026.06.08.731026; this version posted June 10, 2026. The copyright holder for this preprint (which was not certified by peer review) is the author/funder, who has granted bioRxiv a license to display the preprint in perpetuity. It is made available under a CC-BY 4.0 International license.

# Supplementary information

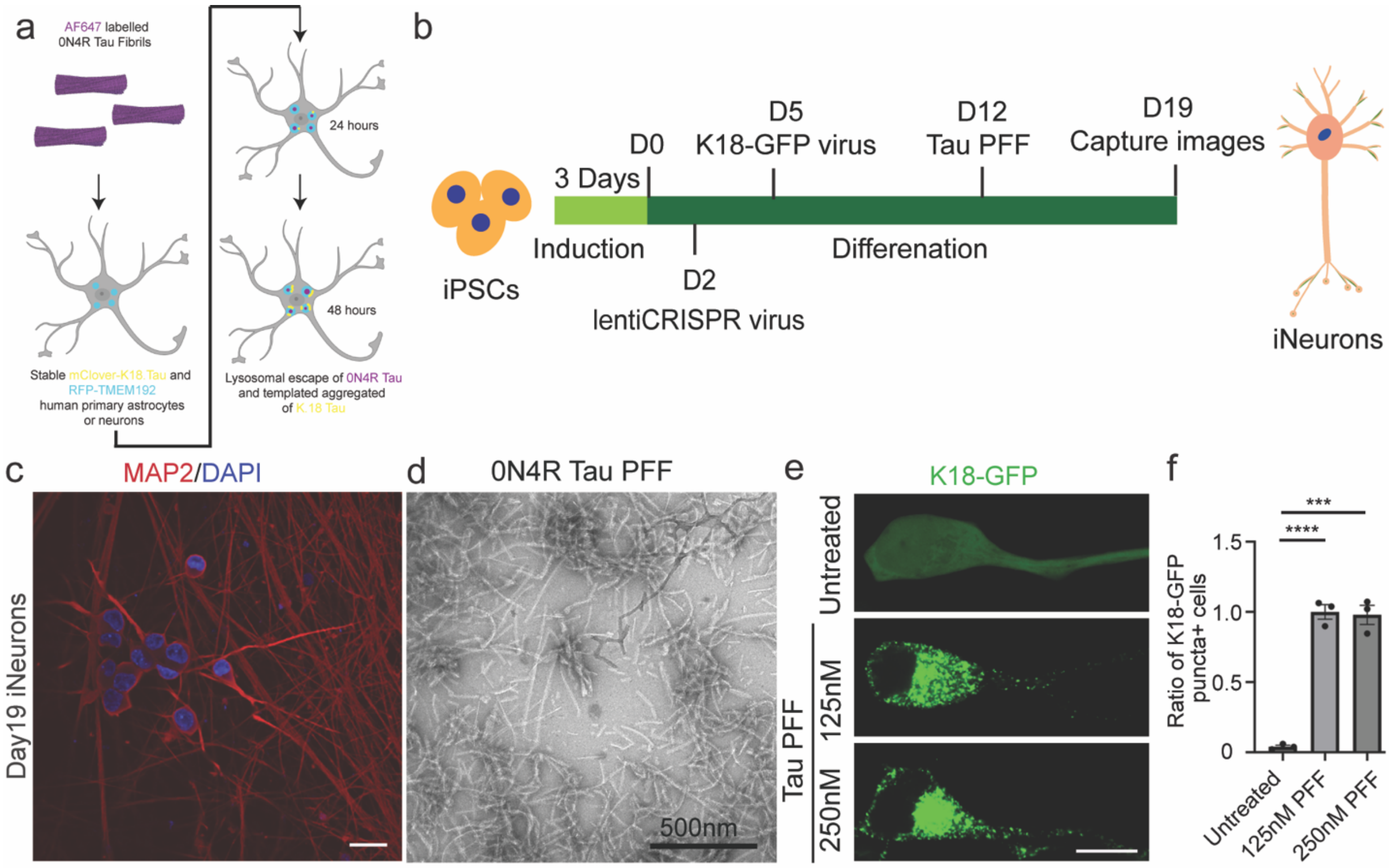


*Figure S1: Establishment of pathogenic templating in iPSC-derived human neurons. (a) Schematic of the K18-GFP/TMEM192-RFP reporter system used to monitor lysosomal breach. (b) Experimental timeline for the maturation of iNeurons and subsequent tau PFF treatment. (c) Immunofluorescence characterization of Day 19 iNeurons positive for the mature neuronal marker MAP2. (d) Negative stain images of Tau PFF prior to sonication (e) Comparison of K18.Tau aggregation following exposure to 125 nM and 250 nM tau PFFs, showing successful induction of fluorescent puncta. (f) Evaluation of (e) showing the ratio of k18.tau puncta per cells. The ratio of K18-GFP puncta+ cells shown in the histogram was normalized to that of the 125nM PFF-treated group. An unpaired two-tailed Student's t-test was used for pairwise comparisons. Scale bars are 20μm.*

bioRxiv preprint doi: https://doi.org/10.64898/2026.06.08.731026; this version posted June 10, 2026. The copyright holder for this preprint (which was not certified by peer review) is the author/funder, who has granted bioRxiv a license to display the preprint in perpetuity. It is made available under a CC-BY 4.0 International license.

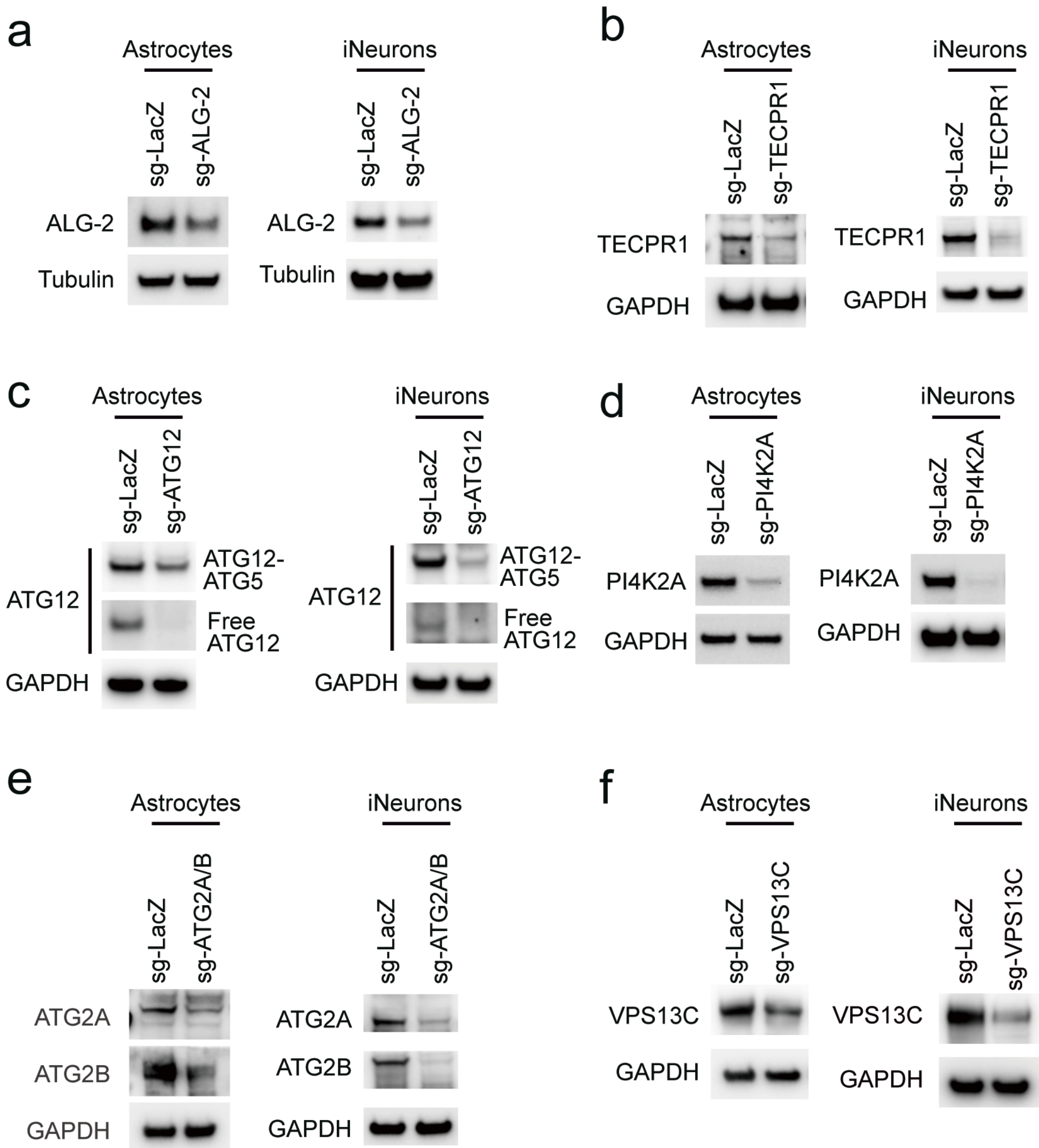


*Figure S2: Knockdown efficiency of the indicated genes in astrocytes and iNeurons. (A). Knockdown efficiency of ALG-2 in astrocytes and iNeurons was assessed by Western blot. (B). Knockdown efficiency of TECPR1 in astrocytes and iNeurons was assessed by Western blot. (C). Knockdown efficiency of ATG12 in astrocytes was assessed by Western blot. (D). Knockdown efficiency of PI4K2A in astrocytes and iNeurons was assessed by Western blot. (E). Knockdown efficiency of ATG2A and ATG2B in astrocytes and iNeurons was assessed by Western blot. (F). Knockdown efficiency of VPS13C in astrocytes and iNeurons was assessed by Western blot.*

bioRxiv preprint doi: https://doi.org/10.64898/2026.06.08.731026; this version posted June 10, 2026. The copyright holder for this preprint (which was not certified by peer review) is the author/funder, who has granted bioRxiv a license to display the preprint in perpetuity. It is made available under a CC-BY 4.0 International license.

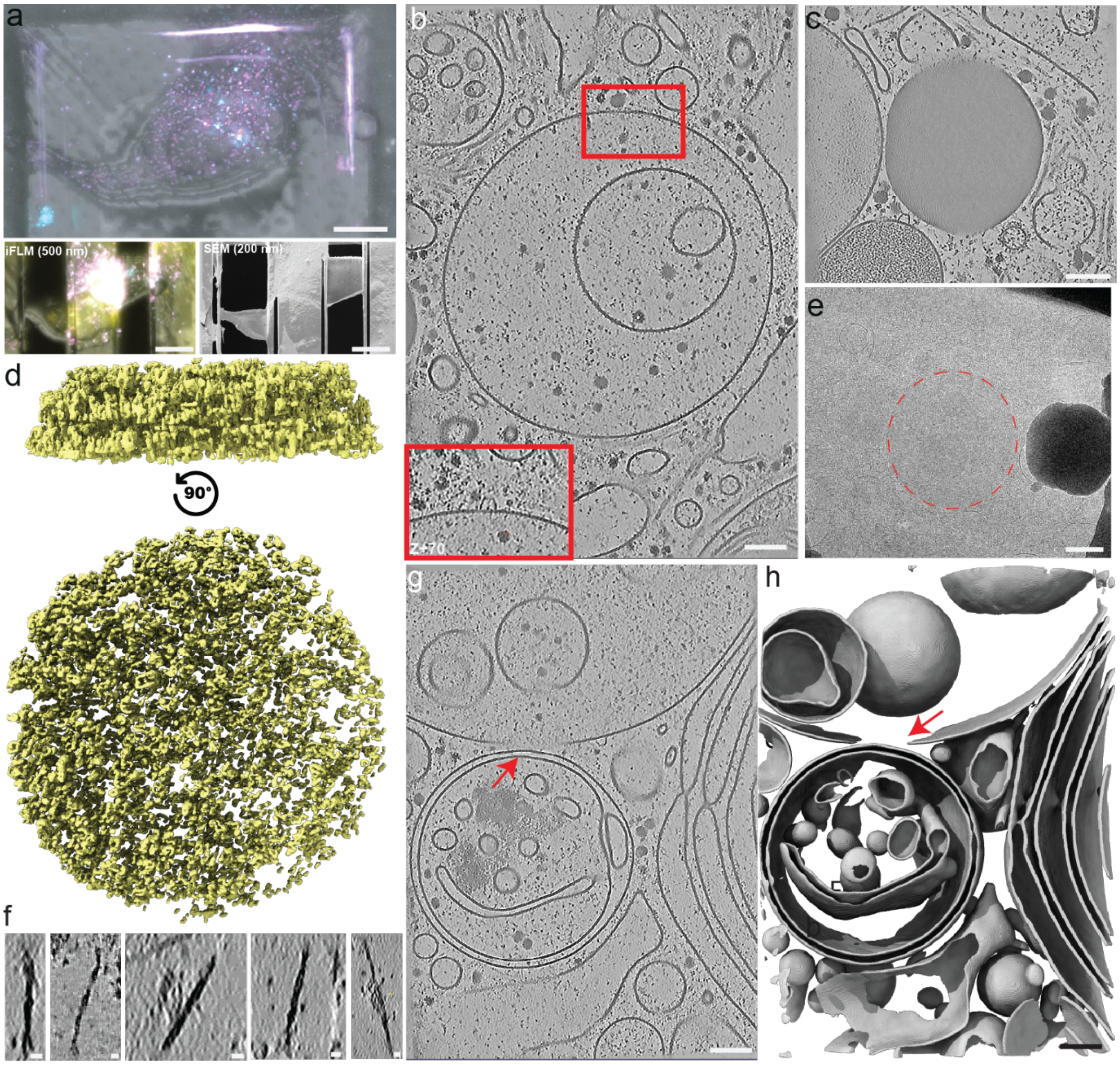


*Figure S3: Correlative Validation of Lysosomes. (a) Representative correlative light microscopy (CLEM) images of FIB-milled lamellae. iFLM images were acquired at 500nm and afterwards milled down to 200 nm thickness. Scale bars are 20 and 10 um. (b) Tomographic slice showing the limiting membrane of a target organelle decorated with flotillin complexes (red box), verifying its identity as a lysosome. Scale bar is 100 nm. (c) Structural comparison between a tau-filled lysosome and a cytoplasmic lipid droplet. Sclae bar is 100 um. (d) Additional gallery of 3D segmented intraluminal tau fragments (yellow) viewed from multiple orientations. (e) Medium magnification view of the full swollen lysosome shown in 4a. Sclae bar is 500 nm. (f) Gallery of cytosolic tau fibrils shown in 5f. scale bar is 10 nm. Representative view of a untreated lysosome with a membrane discontinuity (red arrow) aligning with the tilt axis. Scale bar is 100 nm. (h) Membrane Segmentation of f. Scale bar is 100 nm.*
